# Genetic Variants, Serum 25-Hydroxyvitamin D Levels, and Sarcopenia: A Mendelian Randomization Analysis

**DOI:** 10.1001/jamanetworkopen.2023.31558

**Published:** 2023-08-30

**Authors:** Tingting Sha, Yilun Wang, Yuqing Zhang, Nancy E. Lane, Changjun Li, Jie Wei, Chao Zeng, Guanghua Lei

**Affiliations:** 1 Department of Orthopaedics, Xiangya Hospital, Central South University, Changsha, China; 2 Hunan Key Laboratory of Joint Degeneration and Injury, Changsha, China; 3 Key Laboratory of Aging-related Bone and Joint Diseases Prevention and Treatment, Ministry of Education, Changsha, China; 4 Division of Rheumatology, Allergy, and Immunology, Department of Medicine, Massachusetts General Hospital, Harvard Medical School, Boston; 5 The Mongan Institute, Massachusetts General Hospital, Harvard Medical School, Boston; 6 Center for Musculoskeletal Health and Department of Medicine, University of California School of Medicine, Sacramento; 7 National Clinical Research Center for Geriatric Disorders, Xiangya Hospital, Central South University, Changsha, China; 8 Department of Endocrinology, Endocrinology Research Center, Xiangya Hospital of Central South University, Changsha, China; 9 Health Management Center, Xiangya Hospital, Central South University, Changsha, China; 10 Department of Epidemiology and Health Statistics, Xiangya School of Public Health, Central South University, Changsha, China

## Abstract

**Importance:**

Vitamin D deficiency is commonly associated with sarcopenia; however, the latest International Clinical Practice Guidelines for Sarcopenia do not recommend vitamin D supplementation for sarcopenia owing to a lack of an apparent therapeutic effect on the indices of sarcopenia among participants with replete vitamin D concentration (ie, 25-hydroxyvitamin D [25(OH)D] level >20 ng/mL) from randomized clinical trials. While there is consensus in all vitamin D guidelines that serum levels of 25(OH)D less than 10 ng/mL should be corrected, approximately 30% of the world population’s 25(OH)D levels range from 10 to 20 ng/mL, and it remains unclear whether such suboptimal levels can maintain optimal health, including sarcopenia risk.

**Objective:**

To investigate the association of serum 25(OH)D level, especially suboptimal levels, with sarcopenia risk.

**Design, Setting, and Participants:**

This genome-wide genetic association study was performed from August 2022 to February 2023 among the 295 489 unrelated European participants from the UK Biobank (2006-2010). Nonlinear and standard mendelian randomization analyses were used to examine the association of serum 25(OH)D concentration with sarcopenia risk.

**Exposures:**

A weighted genetic risk score using 35 unrelated single-nucleotide variants from the UK Biobank and weights from the SUNLIGHT Consortium was selected as an instrumental variable for serum 25(OH)D concentration.

**Main Outcomes and Measures:**

The primary outcome was sarcopenia, and the secondary outcomes consisted of grip strength, appendicular lean mass index, and gait speed.

**Results:**

The final genetic analyses included 295 489 participants (mean [SD] age, 56.3 [8.1] years; 139 216 female [52.9%]). There was an L-shaped association between genetically predicted serum 25(OH)D concentration and sarcopenia risk. The risk of sarcopenia decreased rapidly as 25(OH)D concentration increased until 20 ng/mL and then leveled off. The odds ratio of sarcopenia for serum 25(OH)D level of 10 vs 20 ng/mL was 1.74 (95% CI, 1.17-2.59). Similar patterns were also observed when the association between serum 25(OH)D concentration and risks of each of the sarcopenia indices were evaluated.

**Conclusions and Relevance:**

In this mendelian randomization genetic association study of adults in the UK Biobank, the findings supported a nonlinear association between suboptimal 25(OH)D levels and sarcopenia risk. Randomized clinical trials among participants with suboptimal 25(OH)D levels are required to verify the potential causality.

## Key Points

**Question:**

What is the association of suboptimal levels of serum 25-hydroxyvitamin D (25[OH]D) (ie, 10-20 ng/mL) with the risk of sarcopenia?

**Findings:**

In this genetic association study analyzing 295 489 participants using mendelian randomization, an L-shaped association was observed between genetically predicted serum 25(OH)D concentration and the risk of sarcopenia. There was an apparent threshold level of serum 25(OH)D, ie, 20 ng/mL, for the risk of sarcopenia, and above that level, there was no significant association with sarcopenia.

**Meaning:**

If confirmed by randomized clinical trials, these findings suggest that population-wide correction of low vitamin D status could be a cost-effective measure to reduce the burden of sarcopenia.

## Teaser

This genetic association study investigates serum 25-hydroxyvitamin D levels and sarcopenia risk among participants in the UK Biobank.

## Introduction

Sarcopenia is a common skeletal muscle disorder among older adults, with prevalence ranging from 10% to 27% in people aged 60 years and older.^1^ It has been projected that by 2050, approximately 2 billion people worldwide will develop sarcopenia.^2^ Sarcopenia is also associated with an increased risk of various adverse conditions, including impaired mobility, increased risk of morbidity, hospitalization, and mortality.^3^ However, currently, no effective treatments can reverse or alter the progression of sarcopenia.^4^

Vitamin D is a fat-soluble secosteroid that regulates bone health by increasing intestinal absorption of calcium and phosphate. Extensive evidence, mainly from animal studies, suggests that vitamin D directly affects muscle metabolism and may have a role in preventing muscle weakness during aging.^5,6,7^ However, randomized clinical trials (RCTs) of vitamin D monotherapy in patients with sarcopenia have yielded conflicting results. Most RCTs reported a null effect.^8,9,10,11,12,13^ Recently, a meta-analysis of RCTs of vitamin D supplementation reported no overall improvement in any sarcopenia indices.^14^ All RCTs that failed to show beneficial effects of vitamin D supplementation were conducted in individuals with serum 25(OH)D concentration greater than 20 ng/mL (to convert to nmol/L, multiply by 2.496).^8,9,10,11,12,13^ As a result, the latest International Clinical Practice Guidelines for Sarcopenia do not recommend vitamin D supplementation for sarcopenia.^15^

Despite the consensus in all guidelines that serum 25(OH)D levels less than 10 ng/mL should be corrected, the recommended minimal desirable concentration varies widely, ranging from 10 to greater than 40 ng/mL.^16^ Specifically, serum 25(OH)D level of 10 to 20 ng/mL represents an uncertain range for maintaining optimal health.^17^ A previous study reported that approximately 30% of the world’s population had their serum vitamin D levels in this range^18^; however, RCTs targeting this group of people are lacking. Indeed, the presence of a threshold effect has been proposed, where disease risk and benefits of vitamin D supplementation may only surface below certain thresholds (ie, 25[OH]D <20 ng/mL).^19^ Better characterization of the association between low vitamin D status and sarcopenia risk would have significant public health implications for reducing the burden of this disease.

Mendelian randomization (MR) is a technique that uses genetic variants as proxies for the exposure of interest. It offers several advantages over observational studies. First, MR studies are relatively immune to common behavioral, physiological, and socioeconomic confounders owing to random assignment of alleles at meiosis. Second, although reverse causality cannot be completely avoided, the results of MR studies can minimize reverse causality because genetic variants are fixed at conception. Third, in most cases, genetic variants are precisely measured and reported and thus are less susceptible to bias and measurement errors. Thus, it is especially useful in evaluating risk factors of long-term effects.^20^ As such, the MR approach is particularly helpful in observational studies assessing the association between vitamin D and the risk of sarcopenia, as it provides a measure of an individual’s lifetime vitamin D stores and avoids measurement errors from a single measurement of vitamin D. The MR design resembles an RCT and can aid in strengthening causal inferences on the roles of exposures with significantly reduced concerns on ethical, applicability, and financial issues.^21^ Moreover, the MR approach has been recently expanded to allow for assessing nonlinear associations,^22^ which enables investigation of a potential nonlinear association between vitamin D and the risk of sarcopenia among individuals with suboptimal vitamin D levels. In this study, we applied standard and nonlinear MR to investigate the potential associations of serum 25(OH)D level, especially suboptimal levels, and risk of sarcopenia in a population-based cohort: the UK Biobank.

## Methods

### Data Source and Study Population

**Figure 1.**
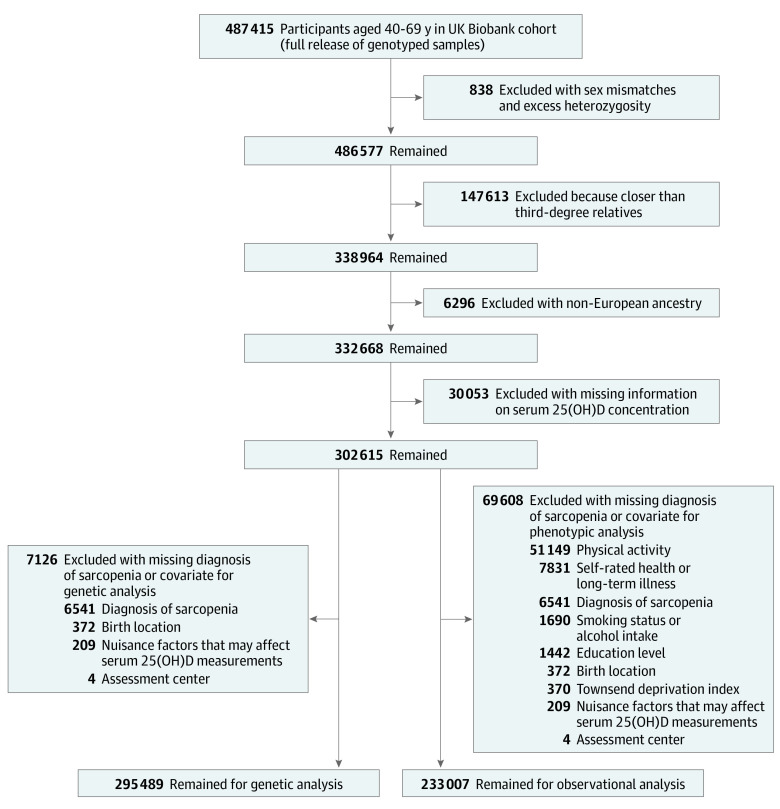
Flowchart of Participant Selection in the UK Biobank 25(OH)D indicates 25-hydroxyvitamin D.

The UK Biobank is a large prospective cohort that recruited more than 500 000 participants aged 40 to 69 years from 22 assessment centers across the UK from 2006 to 2010.^23^ To minimize the potential confounding caused by ancestry, we restricted the analyses to unrelated participants of European ancestry.^24^ The inclusion and exclusion criteria are presented in the eMethods in Supplement 1. Final genetic analyses were conducted among 295 489 participants with complete information on serum 25(OH)D concentration, diagnosis of sarcopenia, and relevant covariates. Detailed information regarding participant selection is available in Figure 1. All cited data sources obtained participant informed consent and relevant ethical approval; therefore, no extra consents or approvals were needed. This study followed the Strengthening the Reporting of Observational Studies in Epidemiology Using Mendelian Randomization (STROBE-MR) reporting guidelines.

### Ascertainment of Exposure and Outcomes

Serum 25(OH)D concentration (ng/mL) was measured using the Liaison XL 25(OH)D assay (DiaSorin). Participants with 25(OH)D concentrations below or above the validated range for the assay were excluded.

The primary outcome was sarcopenia, defined according to the European Working Group on Sarcopenia in Older People in 2019.^25^ The secondary outcomes consisted of 3 indices of sarcopenia, ie, grip strength, appendicular lean mass index, and gait speed. Grip strength was measured using a Jamar J00105 hydraulic handheld dynamometer. Appendicular lean mass was assessed by bioelectrical impedance analysis. Because muscle mass correlates with body size, we calculated appendicular lean mass index (ie, appendicular lean mass/body mass index [calculated as weight in kilograms divided by height in meters squared]).^25^ Gait speed was self-reported with the question, “How would you describe your usual walking speed?”

### Instrumental Variables for Exposure

We constructed a weighted genetic risk score using 35 autosomal single-nucleotide variants (SNVs; formerly single-nucleotide polymorphisms [SNPs]) that were discovered in a recent genome-wide association analysis (GWAS) of serum 25(OH)D concentration in the UK Biobank.^26^ These SNVs had been replicated with a consistent direction and a *P* value less than .05 in an earlier GWAS by the SUNLIGHT Consortium.^27^ The weight was selected from the SUNLIGHT Consortium to avoid bias arising from using internal weights.^28^ Information on these 35 variants can be found in eTable 1 in Supplement 1.

### Statistical Analysis

Statistical analyses were performed from August 2022 to February 2023. We first examined the association of 25(OH)D concentration with sarcopenia indices by fitting logistic or linear regression models. In the multivariable-adjusted regression models, we adjusted for demographic, lifestyle, general health, and socioeconomic factors as well as several other factors that may affect serum 25(OH)D measurements.^6,7^ We used fractional polynomial models to investigate the shape of the association between serum 25(OH)D concentration and each of the sarcopenia indices.^29^

We then computed the MR estimates within categories of residual 25(OH)D concentration (<10.0 ng/mL, 10.0-19.9 ng/mL, 20.0-29.9 ng/mL, and ≥30.0 ng/mL) using the ratio-of-coefficients method to assess the association between the genetically predicted 25(OH)D concentration and sarcopenia. For genetic analyses, we adjusted for age, age squared, sex, assessment centers, top 20 principal components of ancestry, genotyping arrays, and nuisance factors that might affect serum 25(OH)D measurements. To avoid collider bias, the stratification was performed using residual 25(OH)D concentration rather than raw 25(OH)D values.^30^ Furthermore, to account for potential horizontal pleiotropy in the standard MR model, 4 MR approaches (ie, inverse-variance weighted, weighted median, MR-Egger, and MR Pleiotropy RESidual Sum and Outlier [MR-PRESSO]) (eMethods in Supplement 1) were used to test the associations between the 25(OH)D concentration and sarcopenia indices. Finally, we performed RadialMR analyses using modified second-order weights to identify outliers.^31^

Given that the shape of the association between 25(OH)D concentration and sarcopenia was nonlinear in the observational analysis, we performed a nonlinear MR analysis with the fractional polynomial method to examine the nonlinearity of the exposure-outcome association (eMethods in Supplement 1).^22^ Further, considering that advancing age may alter vitamin D levels and increase the risk of developing sarcopenia, we performed an age-stratified nonlinear MR analysis to investigate whether the association between 25(OH)D concentration and sarcopenia risk varied according to age stratum.^32^

The validity of causal inferences drawn from MR relies on 3 key assumptions (eMethods in Supplement 1). We took several steps to assess the potential violation of MR assumptions, which are described in further detail in the eMethods in Supplement 1. We used the odds ratios (ORs) or betas (βs) as the estimates for the association of serum 25(OH)D concentration with the risk of sarcopenia or its related indices, respectively. The *P* values obtained were 2-tailed for all statistical tests. Analyses were conducted in R software, version 4.1.2 (R Foundation for Statistical Computing).

## Results

### Baseline Characteristics

A total of 295 489 participants (mean [SD] age, 56.3 [8.1] years; 139 216 female [52.9%]) of unrelated European ancestry from the UK Biobank were included in the genetic analyses. Of these, 233 007 participants were included in the further analyses. The baseline characteristics in the observational analyses are shown in Table 1.

**Table 1.** Participant Characteristics at Recruitment in the Observational Analysis

Characteristic	Participants, No. (%)			
	Serum 25(OH)D, ng/mL			
	<10.0 (n = 29 887)	10.0-19.9 (n = 96 464)	20.0-29.9 (n = 79 432)	≥30.0 (n = 27 224)
Age, mean (SD), y	54.3 (8.1)	55.8 (8.1)	56.9 (8.0)	56.9 (8.1)
Sex				
Female	14 937 (50.0)	48 889 (50.7)	40 840 (51.4)	13 984 (51.4)
Male	14 950 (50.0)	47 575 (49.3)	38 592 (48.6)	13 240 (48.6)
BMI, mean (SD)	28.3 (5.6)	27.7 (4.8)	26.7 (4.2)	25.8 (3.8)
Townsend deprivation index	−0.5 (3.4)	−1.3 (3.1)	−1.8 (2.8)	−1.9 (2.7)
Smoking status				
Nonsmoker	16 051 (53.7)	53 473 (55.4)	44 139 (55.6)	14 669 (53.9)
Previous smokers	9155 (30.6)	33 135 (34.4)	28 906 (36.4)	10 324 (37.9)
Current smokers	4681 (15.7)	9856 (10.2)	6387 (8.0)	2231 (8.2)
Alcohol intake				
Nondrinkers	2179 (7.3)	3711 (3.9)	2124 (2.7)	568 (2.1)
Previous drinkers	1453 (4.9)	3285 (3.4)	2212 (2.8)	778 (2.9)
Current drinkers	26 255 (87.8)	89 468 (92.7)	75 096 (94.5)	25 878 (95.1)
Physical activity				
Light	7925 (26.5)	19 523 (20.2)	12 154 (15.3)	3409 (12.5)
Moderate	12 839 (43.0)	41 039 (42.5)	31 874 (40.1)	9827 (36.1)
Vigorous	9123 (30.5)	35 902 (37.2)	35 404 (44.6)	13 988 (51.4)
Education level				
NVQ/CSE/A levels/other professional qualifications	16 399 (54.9)	52 321 (54.2)	40 713 (51.3)	13 042 (47.9)
College or university degree	13 488 (45.1)	44 143 (45.8)	38 719 (48.7)	14 182 (52.1)
Self-rated health				
Excellent	4105 (13.7)	16 833 (17.5)	16 336 (20.6)	6294 (23.1)
Good	16 044 (53.7)	56 375 (58.4)	48 185 (60.7)	16 306 (59.9)
Fair	7578 (25.4)	19 511 (20.2)	12 950 (16.3)	3941 (14.5)
Poor	2160 (7.2)	3745 (3.9)	1961 (2.5)	683 (2.5)
Long-term illness				
No	19 075 (63.8)	66 375 (68.8)	56 793 (71.5)	19 390 (71.2)
Yes	10 812 (36.2)	30 089 (31.2)	22 639 (28.5)	7834 (28.8)
Fasting time, mean (SD), h	4.1 (2.9)	3.8 (2.4)	3.7 (2.2)	3.7 (2.2)
Calendar month of assessment visit, mean (SD)	5.0 (3.5)	6.1 (3.4)	6.9 (3.0)	7.3 (2.7)
Sample aliquots for measurement, mean (SD)	6.3 (0.4)	6.3 (0.4)	6.2 (0.4)	6.2 (0.4)

Abbreviations: BMI, body mass index, calculated as weight in kilograms divided by height in meters squared; CSE, Certificate of Secondary Education; NVQ, National Vocational Qualification.

SI conversion factor: To convert ng/mL to nmol/L, multiply by 2.496.

### Observational Analyses

**Figure 2.**
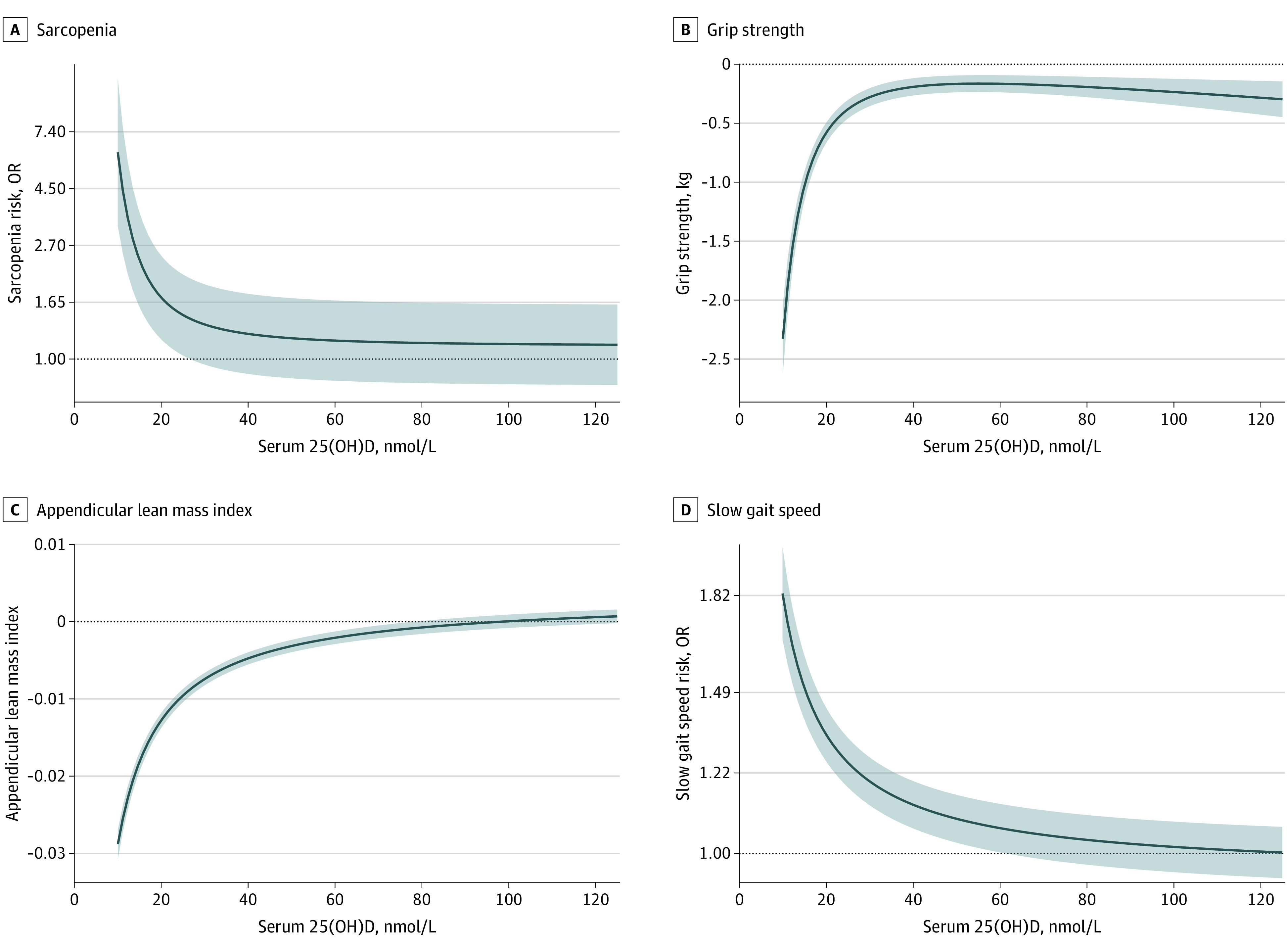
Phenotypic Associations of Serum 25-Hydroxyvitamin D (25[OH]D) With Risk of Sarcopenia Traits Phenotypic associations of serum 25(OH)D concentration with sarcopenia (A), grip strength (B), appendicular lean mass index (C), and slow gait speed (D). A total of 233 007 participants were included in the observational analyses for sarcopenia, grip strength, and appendicular lean mass index, and 232 271 participants for slow gait speed. To convert nmol/L to ng/mL, divide by 2.496. Shaded areas indicate 95% CIs; OR, odds ratio.

Serum 25(OH)D levels below 20 ng/mL adjusted for multivariable factors were inversely associated with each of the sarcopenia indices (Table 2). Compared with the serum 25(OH)D 20.0 to 29.9 ng/mL category, the lowest 25(OH)D concentrations (ie, <10.0 ng/mL) were associated with a higher OR for sarcopenia (OR, 1.52; 95% CI, 1.09-2.13; *P* = .01) and slow gait speed (OR, 1.32; 95% CI, 1.24-1.40; *P* < .001), as well as the lower levels of grip strength (β = −0.51; 95% CI, −0.61 to −0.41; *P* < .001) and appendicular lean mass index (β = −0.012; 95% CI, −0.013 to −0.011; *P* < .001). Similar results were observed for the 3 sarcopenia indices with serum 25(OH)D concentrations of 10 to 19.9 ng/mL. There was an L-shaped dose-response association between serum 25(OH)D concentration and each of the sarcopenia indices (Figure 2).

**Table 2.** Association Between Serum 25-Hydroxyvitamin D (25[OH]D) Level of Each Category and the Risk of Sarcopenia Indices

	Serum 25(OH)D, ng/mL			
	<10.0 (n = 29 887)	10.0-19.9 (n = 96 464)	20.0-29.9 (n = 79 432)	≥30.0 (n = 27 224)
Sarcopenia				
Event, No.	84	125	107	50
Crude OR (95% CI)	2.09 (1.57 to 2.78)	0.96 (0.74 to 1.25)	1 [Reference]	1.36 (0.98 to 1.91)
Adjusted OR (95% CI)	1.52 (1.09 to 2.13)	1.04 (0.79 to 1.37)	1 [Reference]	1.06 (0.75 to 1.51)
Grip strength				
Mean (SD)	33.4 (11.3)	33.8 (11.3)	33.7 (11.3)	33.60 (11.1)
Crude β (95% CI)	−0.22 (−0.37 to −0.07)	0.17 (0.06 to 0.27)	0 [Reference]	−0.05 (−0.21 to 0.10)
Adjusted β (95% CI)	−0.51 (−0.61 to −0.41)	−0.06 (−0.13 to 0.01)	0 [Reference]	−0.06 (−0.16 to 0.04)
Appendicular lean mass index				
Mean (SD)	0.8 (0.2)	0.8 (0.2)	0.8 (0.2)	0.9 (0.2)
Crude β (95% CI)	−0.018 (−0.019 to −0.015)	−0.008 (−0.010 to −0.007)	0 [Reference]	0.009 (0.007 to 0.012)
Adjusted β (95% CI)	−0.012 (−0.013 to −0.011)	−0.004 (−0.005 to −0.003)	0 [Reference]	0.001 (−0.001 to 0.002)
Slow gait speed				
Event, No.	3270	6757	3733	1176
Crude OR (95% CI)	2.50 (2.38 to 2.63)	1.53 (1.47 to 1.59)	1 [Reference]	0.92 (0.86 to 0.98)
Adjusted OR (95% CI)	1.32 (1.24 to 1.40)	1.14 (1.09 to 1.20)	1 [Reference]	1.08 (0.99 to 1.16)

Abbreviation: OR, odds ratio.

SI conversion factor: To convert ng/mL to nmol/L, multiply by 2.496.

### Standard MR Analyses

In the stratified MR analyses, the results only support an association of genetically predicted 25(OH)D concentration with sarcopenia (OR, 1.01; 95% CI, 1.00-1.02; *P* = .03) in the category of the lowest 25(OH)D concentrations (eTable 2 in Supplement 1). Similar patterns were also observed among the individuals with the lowest or suboptimal serum 25(OH)D levels. Using the other 4 MR methods (eTable 3 in Supplement 1), we found no significant association between 25(OH)D concentration and sarcopenia indices. The MR-PRESSO and MR-Egger intercepts indicated limited evidence of pleiotropy. After excluding the outliers detected by RadialMR analyses, the MR estimates did not change markedly (eFigure 1 and eTable 4 in Supplement 1).

### Nonlinear MR Analyses

**Figure 3.**
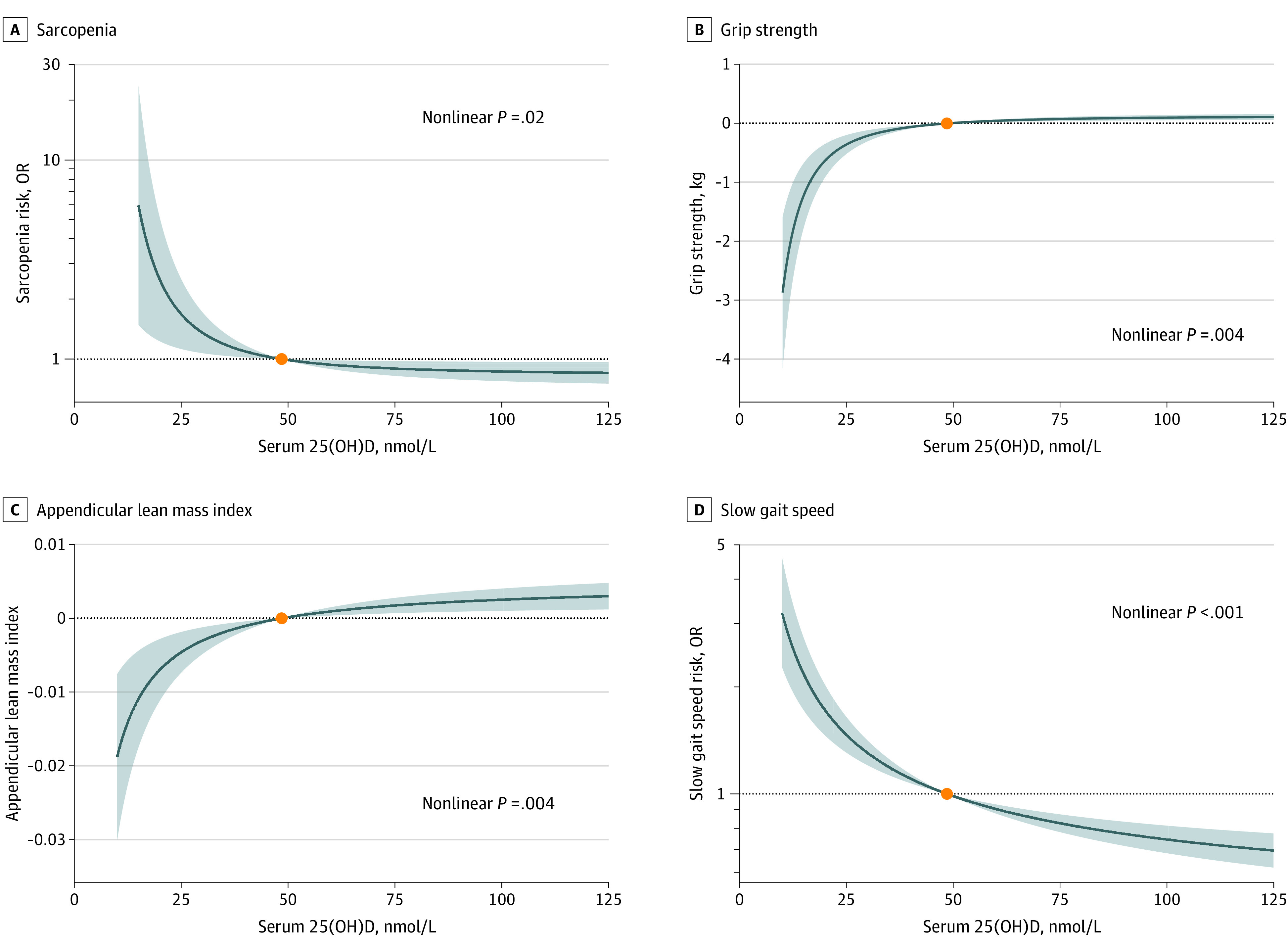
Genetic Associations of Serum 25-Hydroxyvitamin D (25[OH]D) With Risk of Sarcopenia Using 35 Single-Nucleotide Variants to the Instrument Genetic associations of serum 25(OH)D concentration with sarcopenia (A), grip strength (B), appendicular lean mass index (C), and slow gait speed (D). The orange dot represents the reference point of serum 25(OH)D of 50 nmol/L (to convert to ng/mL, divide by 2.496). The adjustment included age, age squared, sex, assessment center, birth location, top 20 genetic principal components, genotyping array in both stages, and nuisance factors that could affect serum 25(OH)D measurements, including the month when the blood sample was taken, fasting time before the blood sample was taken, and sample aliquots for measurement. A total of 295 489 participants were included in the genetic analyses for sarcopenia, grip strength, and appendicular lean mass index, and 293 119 participants for slow gait speed. Shaded areas indicate 95% CIs; OR, odds ratio.

As shown in Figure 3, there was an L-shaped association between genetically predicted serum 25(OH)D concentration and the risk of sarcopenia (Figure 3A, nonlinear *P* = .02). The OR of sarcopenia decreased sharply with increasing 25(OH)D concentration for participants when serum 25(OH)D concentration was less than 20 ng/mL. And such an association disappeared when 25(OH)D concentration was greater than 20 ng/mL, indicating an apparent threshold near 20 ng/mL of 25(OH)D concentration. The OR of sarcopenia for serum 25(OH)D level of 10 vs 20 ng/mL was 1.74 (95% CI, 1.17-2.59). There appeared to be a slight further lowering in the odds of sarcopenia with higher concentrations. For example, the OR of sarcopenia for serum 25(OH)D level of 30 vs 20 ng/mL was 0.89 (95% CI, 0.82-0.97).

We observed an inverse L-shaped association of genetically predicted serum 25(OH)D concentration with grip strength (Figure 3B, nonlinear *P* = .004) and appendicular lean mass index (Figure 3C, nonlinear *P* = .004). There was a progressive increase in the grip strength and appendicular lean mass index as serum 25(OH)D concentration when serum 25(OH)D concentration was below 20 ng/mL. Then the associations plateaued, suggesting a threshold effect near 20 ng/mL. Similar patterns were also observed when the association between genetically predicted serum 25(OH)D levels and the risk of slow gait speed was evaluated (Figure 3D, nonlinear *P* < .001).

### Age-Stratified Nonlinear MR Analyses

eFigure 2 in Supplement 1 shows the results stratified by age strata. The nonlinear association between genetically predicted serum 25(OH)D concentration and sarcopenia risk was observed in both the younger than 65 years and the 65 years and older age groups. In the younger than 65 years age group, there was a significant L-shaped association for sarcopenia (nonlinear *P* = .05) and slow gait speed (nonlinear *P* = .02), as well as inverse L-shaped associations for genetically predicted grip strength (nonlinear *P* = .003) and appendicular lean mass index (nonlinear *P* = .03) (eFigure 2 in Supplement 1). In the 65 years and older age group, nonlinear associations were also observed between genetically predicted serum 25(OH)D concentration and risks of sarcopenia (nonlinear *P* = .05), gait speed (nonlinear *P* < .001) and appendicular lean mass index (nonlinear *P* = .05) (eFigure 2 in Supplement 1). However, there was no apparent evidence for the nonlinear association between 25(OH)D and grip strength in the 65 years and older age group (nonlinear *P* > .99) (eFigure 2 in Supplement 1).

### Sensitivity Analyses to Verify MR Assumptions and Evaluate Bias

We found little evidence that vitamin D genetic risk score was associated with potential confounders, including body mass index, smoking, alcohol intake, physical activity, and Townsend deprivation index (eTable 5 in Supplement 1). The various sensitivity analyses showed similar L-shaped associations of genetically predicted 25(OH)D concentration with sarcopenia and its indices. Detailed results of sensitivity analyses are presented in eResults, eTables 6 and 7, and eFigure 3 in Supplement 1.

## Discussion

Using MR analysis, we found an apparent L-shaped association between serum 25(OH)D concentration and sarcopenia risk. The risk of sarcopenia was the highest among individuals with the lowest vitamin D concentration and leveled off at about 20 ng/mL. Similar patterns were observed when associations between serum 25(OH)D levels and risks of each of the sarcopenia indices were evaluated. These findings strongly support the previously proposed threshold effect of serum 25(OH)D concentration on sarcopenia.^19^

Numerous observational studies have examined the association between serum vitamin D levels and sarcopenia risk. The results suggest that low vitamin D status, ie, 25(OH)D less than 20 ng/mL, is associated with an increased sarcopenia risk,^33^ reduced muscle strength and muscle mass,^34^ and impaired physical performance.^35^ However, the results from RCTs were inconclusive. Of 7 RCTs that examined the effect of vitamin D supplementation on indices of sarcopenia,^8,9,10,11,12,13,36^ 6 failed to show any benefits.^8,9,10,11,12,13^ All 6 RCTs were conducted among the participants with 25(OH)D levels greater than 20 ng/mL.^8,9,10,11,12,13^ Interestingly, in the 1 study that demonstrated a beneficial effect of vitamin D vs placebo on sarcopenia risk in postmenopausal women, 25(OH)D levels were between 15 and 17 ng/mL.^36^ Even so, trials of vitamin D supplementation can be compounded by contamination of the placebo group and unblinding, given that vitamin D supplementation and testing are easily accessible.

The association of vitamin D levels with sarcopenia or its related indices has previously been investigated in 4 MR studies.^37,38,39,40^ Three of them used the standard MR analysis.^38,39,40^ One study reported a null association between 25(OH)D and sarcopenia risk,^38^ and the other 2 showed a beneficial effect of 25(OH)D on grip strength^39^ and lean mass.^40^ However, these studies only used 6 SNVs as instruments to infer serum 25(OH)D levels, which might be hindered by the low power to predict low serum 25(OH)D concentration.^39^ Our study used up to 35 replicated SNVs, thereby allowing the robustness of the GWAS signals and much larger variations in serum 25(OH)D concentration to be predicted. Our analysis indicated no apparent linear associations of 25(OH)D with sarcopenia and its indices. These findings are consistent with the reports from the earlier RCTs.^8,9,10,11,12,13^ Of the 4 MR analyses, 1 examined the nonlinear association between 25(OH)D and grip strength and failed to demonstrate an apparent nonlinear association.^37^ However, the study did not exclude related participants; as such, the potential confounding caused by ancestry might dilute the association.^41^ In our study, we only included unrelated participants of European ancestry; thus, the findings are compatible with the true association between vitamin D level and sarcopenia risk, mainly observed among those with low vitamin D status.

The association between low vitamin D status and sarcopenia risk is biologically plausible. It was reported that muscle atrophy, histological changes, and muscle weakness independent of secondary metabolic changes were observed in animal models with vitamin D deficiency or impaired vitamin D utilization (ie, vitamin D receptor knockout mice).^5^ Such effects can be attributed to 2 mechanisms: short-term/long-term responses in skeletal muscle involving the genomic mode and the nongenomic mode of action. Specifically, the genomic pathway is more classical. In this mechanism, secosteroid hormone induces the differentiation and proliferation of muscle cells through nuclear vitamin D receptor–mediated gene transcription in myoblasts, resulting in the growth of skeletal muscle fibers.^42^ In the nongenomic pathway, vitamin D may play a role in growth-related signal transduction and rapid regulation of the calcium messenger system, thereby affecting the contraction of skeletal muscle.^43^ Moreover, vitamin D level has also been recognized as an influencing factor for inflammation, insulin secretion, and sensitivity, all factors that have been associated with sarcopenia.^44^

Using a large population-based database, we assessed the associations between serum 25(OH)D level and the risk of sarcopenia and its 3 indices. The MR approach allowed us to explore the association between the suboptimal serum 25(OH)D levels and the risk of sarcopenia that previous RCTs have not assessed. Additionally, we performed a nonlinear MR analysis to characterize the shape of the association between genetically predicted serum 25(OH)D concentration and sarcopenia risk. We found an apparent threshold level of serum 25(OH)D, ie, 20 ng/mL, for the risk of sarcopenia, and above that level, there was no significant additional protective association with sarcopenia. Despite the impossibility of proving the validity of MR assumptions,^45^ we implemented several strategies to identify potential violations in the assumptions and gauge our findings’ robustness, and no evidence of violations was found. Moreover, the robustness of the L-shaped association between genetically predicted serum 25(OH)D concentration and sarcopenia risk was confirmed through various sensitivity analyses.^26^

### Limitations

Some limitations are noteworthy. First, we restricted our analysis to participants of White British descent. While minimizing bias due to population stratification, it may limit the transferability of our findings to other racial and ethnic groups. Second, with only a 5% response rate, UK Biobank is not representative of the general public in the UK despite its large sample size. It is uncertain to what extent this selection could affect our MR analyses. Finally, genetic instruments approximate the average effects over the life course, as with all MR studies. Thus, the true shape and strength of the biological association between serum vitamin D level and sarcopenia risk could be more complex than that presented in our study.

To date, all available vitamin D guidelines unanimously agree that serum levels of 25(OH)D less than 10 ng/mL should be corrected; however, 10 to 20 ng/mL represents an uncertain range and can be sufficient or not for specific individuals.^17^ Approximately 30% of the world population had serum 25(OH)D concentrations that ranged from 10 to 20 ng/mL,^18^ and 41.7% of the participants in our study fell in this range. As participants in the UK Biobank are generally healthier than the general public, the true prevalence of such vitamin D status in the UK population is likely to be higher. Nevertheless, RCTs recruiting patients with sarcopenia with 25(OH)D levels in this range are still lacking. Our study showed that individuals with 25(OH)D levels at 10 ng/mL had 74% higher odds of sarcopenia compared with 20 ng/mL, indicating important public health implications. If confirmed by RCTs, our findings suggest that population-wide correction of low vitamin D status could be a cost-effective measure to reduce the burden of sarcopenia.

## Conclusions

In this genetic association study, findings support a nonlinear association between suboptimal 25(OH)D levels and sarcopenia risk. Randomized clinical trials among participants with suboptimal 25(OH)D levels are required to verify the potential causality.

## Notes

Supplement 1.
eMethods.

eResults.
**eTable 1.** Genome-wide significant vitamin D variants used for the genetic instruments for serum 25(OH)D concentrations **eTable 2.** Standard linear Mendelian randomization estimates for the associations of genetically predicted serum 25(OH)D with sarcopenia and its indices using 35 SNVs to instrument **eTable 3.** Two-sample Mendelian randomization estimates for the association of genetically predicted serum 25(OH)D with sarcopenia and its indices using 35 SNVs to instrument **eTable 4.** Two-sample Mendelian randomization estimates for the association of genetically predicted serum 25(OH)D with sarcopenia and its indices using 35 SNVs to the instrument after excluding outliers **eTable 5.** Association of vitamin D-GS with potential confounders in the UK Biobank **eTable 6.** Functional blocks-related traits used in the sensitivity analyses **eTable 7.** Sensitivity analysis for non-linear Mendelian randomization analysis of serum 25(OH)D on sarcopenia risk **eFigure 1.** Radial Mendelian randomization plots for serum 25(OH)D and sarcopenia and its indices using 35 SNVs to the instrument **eFigure 2.** Age-stratified non-linear Mendelian randomization analyses of genetic associations of serum 25(OH)D with sarcopenia and its indices, (A) and (E) sarcopenia, (B) and (F) grip strength, (C) and (G) appendicular lean mass index, and (D) and (H) slow gait speed **eFigure 3.** Genetic associations of serum 25(OH)D with sarcopenia and its indices using 122 SNVs to instrument, (A) sarcopenia, (B) grip strength, (C) appendicular lean mass index, and (D) slow gait speed
eReferences

Supplement 2.
Data Sharing Statement
