# Supplementary material for: Genetic Variants, Serum 25-Hydroxyvitamin D Levels, and Sarcopenia: A Mendelian Randomization Analysis
Source: JAMA Netw Open. 2023 Aug 30;6(8):e2331558. doi: 10.1001/jamanetworkopen.2023.31558 (PMC10469287; doi:10.1001/jamanetworkopen.2023.31558)
Supplement: Supplement 1. — eMethods. eResults. eTable 1. Genome-wide significant vitamin D variants used for the genetic instruments for serum 25(OH)D concentrations eTable 2. Standard linear Mendelian randomization estimates for the associations of genetically predicted serum 25(OH)D with sarcopenia and its indices using 35 SNVs to instrument eTable 3. Two-sample Mendelian randomization estimates for the association of genetically predicted serum 25(OH)D with sarcopenia and its indices using 35 SNVs to instrument eTable 4. Two-sample Mendelian randomization estimates for the association of genetically predicted serum 25(OH)D with sarcopenia and its indices using 35 SNVs to the instrument after excluding outliers eTable 5. Association of vitamin D-GS with potential confounders in the UK Biobank eTable 6. Functional blocks-related traits used in the sensitivity analyses eTable 7. Sensitivity analysis for non-linear Mendelian randomization analysis of serum 25(OH)D on sarcopenia risk eFigure 1. Radial Mendelian randomization plots for serum 25(OH)D and sarcopenia and its indices using 35 SNVs to the instrument eFigure 2. Age-stratified non-linear Mendelian randomization analyses of genetic associations of serum 25(OH)D with sarcopenia and its indices, (A) and (E) sarcopenia, (B) and (F) grip strength, (C) and (G) appendicular lean mass index, and (D) and (H) slow gait speed eFigure 3. Genetic associations of serum 25(OH)D with sarcopenia and its indices using 122 SNVs to instrument, (A) sarcopenia, (B) grip strength, (C) appendicular lean mass index, and (D) slow gait speed eReferences [file jamanetwopen-e2331558-s001.pdf]

Supplemental Online Content

Sha T, Wang Y, Zhang Y, et al. Genetic variants, serum 25-hydroxyvitamin D levels, and sarcopenia: a mendelian randomization analysis. *JAMA Netw Open.* 2023;6(8):e2331558. doi:10.1001/jamanetworkopen.2023.31558

eMethods.

eResults.

eTable 1. Genome-wide significant vitamin D variants used for the genetic instruments for serum 25(OH)D concentrations

eTable 2. Standard linear Mendelian randomization estimates for the associations of genetically predicted serum 25(OH)D with sarcopenia and its indices using 35 SNVs to instrument

eTable 3. Two-sample Mendelian randomization estimates for the association of genetically predicted serum 25(OH)D with sarcopenia and its indices using 35 SNVs to instrument

eTable 4. Two-sample Mendelian randomization estimates for the association of genetically predicted serum 25(OH)D with sarcopenia and its indices using 35 SNVs to the instrument after excluding outliers

eTable 5. Association of vitamin D-GS with potential confounders in the UK Biobank

eTable 6. Functional blocks-related traits used in the sensitivity analyses

eTable 7. Sensitivity analysis for non-linear Mendelian randomization analysis of serum 25(OH)D on sarcopenia risk

eFigure 1. Radial Mendelian randomization plots for serum 25(OH)D and sarcopenia and its indices using 35 SNVs to the instrument

eFigure 2. Age-stratified non-linear Mendelian randomization analyses of genetic associations of serum 25(OH)D with sarcopenia and its indices, (A) and (E) sarcopenia, (B) and (F) grip strength, (C) and (G) appendicular lean mass index, and (D) and (H) slow gait speed

eFigure 3. Genetic associations of serum 25(OH)D with sarcopenia and its indices using 122 SNVs to instrument, (A) sarcopenia, (B) grip strength, (C) appendicular lean mass index, and (D) slow gait speed

eReferences

This supplemental material has been provided by the authors to give readers additional information about their work.

© 2023 Sha T et al. *JAMA Network Open.*

eMethods.

The inclusion criteria and exclusion criteria in the present study

The inclusion criteria in the genetic and observational analyses for the present study were: 1) unrelated European ancestry; 2) individuals having complete data regarding serum 25(OH)D concentration and relevant covariates; and 3) having any data on sarcopenia status. The exclusion criteria were: 1) sex mismatches, excess heterozygosity; 2) closer than third-degree relatives; 3) non-European ancestry; 4) missingness of serum 25(OH)D concentration, 5) lack of relevant covariates for genetic and observational analyses; 6) having no data of diagnosis of sarcopenia.

Standard Mendelian randomization (MR) methods

The MR-Egger approach can detect and correct directional pleiotropy by the MR-Egger intercept, albeit it has relatively low statistical.[1] MR Pleiotropy RESidual Sum and Outlier (MR-PRESSO) can help identify and correct horizontal pleiotropic outliers.[2] We also inspected Cochran's Q statistic to assess heterogeneity between individual genetic variants, an indication of the presence of invalid instruments.[3]

Non-linear MR

Non-linear MR is a genetic risk score (GS)-based single-sample approach. In brief, the entire UK Biobank sample was stratified into 100 strata according to the residual variation of the serum 25(OH)D concentration after regressing on the GS. Then, we used the ratio-of-coefficients method to calculate the localized average causal effect (LACE) in each stratum. Further, meta-regression of LACE against the stratum-specific mean exposure was performed by fitting a range of fractional polynomial exposure-outcome models of Degrees 1 and 2. The best-fitting model was identified by the likelihood ratio test, and the polynomial test for non-linearity in which the best-fitting fractional polynomial model of Degree 1 was compared against the linear model was reported.[4]

MR key assumptions

The validity of causal inferences drawn from MR relies on three key assumptions: (1) genetic variants are robustly associated with the exposure (instrument strength), (2) genetic variants are not associated with potential confounders (independence), and (3) genetic variants are associated with the outcome only via the exposure (no unbalanced horizontal pleiotropy).

Several steps to assess the potential violation of MR assumptions

To minimize the potential violation of MR assumptions, we took several steps to assess the validity of the genetic variants. First, we evaluated instrument strength using the F statistic, with an F-value greater than 10 indicating a strong instrument with a low potential for instrument bias.[5] Second, for the independence assumption, we restricted analyses to self-reported European ancestry and adjusted for principal components to minimize the potential confounding by population stratification. Third, to evaluate the independence assumption, we excluded blood, metabolic, renal, and other traits associated variants in the original vitamin D-GS from the PhenoScanner search and re-generated GS for vitamin D independent of these traits. Forth, we examined associations between the Vitamin D-GS and potential confounders to explore the violation of the no horizontal pleiotropy assumptions. Finally, we used a broader set of SNVs that consist of 122 autosomal variants as instrumental variable to serum 25(OH)D concentration to test the robustness of the results of our non-linear MR analyses.

© 2023 Sha T et al. *JAMA Network Open.*

eResults.

Sensitivity analyses to verify MR assumptions and evaluate bias

Our primary vitamin D-GS was robustly associated with serum 25(OH)D concentration in the UK Biobank, explaining 2.1% of the variation (F statistic = 6,139, P<.001). We found little evidence that vitamin D-GS was associated with potential confounders in the UK Biobank, including BMI, smoking, alcohol intake, physical activity, and Townsend deprivation index (P>.05 for all, see **eTable 5**). For the sensitivity analyses, we re-generated vitamin D-GS by excluding blood, metabolic, renal, or other traits-associated variants, separately, and repeated the non-linear MR analyses. The pleiotropic effects of these outliers identified by the PhenoScanner database are shown in **eTable 6**. The L-shaped associations of genetically-predicted 25(OH)D with sarcopenia and its indices were consistent and were not driven by a particular group of variants (**eTable 7**). In addition, vitamin D GS with 122 variants as an instrument provided similar results, confirming the L-shaped associations of genetically-predicted 25(OH)D with sarcopenia and its indices (**eFigure 3**).

© 2023 Sha T et al. *JAMA Network Open.*

eTable 1. Genome-wide significant vitamin D variants used for the genetic instruments for serum 25(OH)D concentrations

No	SNV	Chr	BP	Effect allele	Other allele	EAF	UK Biobank*			SUNLIGHT Consortium†			35 SNVs for vitamin D-GS	122 SNVs for vitamin D-GS
							Beta	SE	P	Beta	SE	P		
1	rs6671730[a]	1	2339139	G	A	0·566	0·0148	0·0020	1·92E-13	0·0061	0·0023	6·65E-03	√	√
2	rs35408430	1	17560195	C	T	0·658	0·0215	0·0021	1·36E-24	0·0237	0·0056	2·72E-05	√	√
3	rs7522116	1	41835685	C	T	0·434	0·0135	0·0020	2·97E-11	0·0117	0·0054	3·08E-02	√	√
4	rs7528419	1	109817192	G	A	0·225	0·0197	0·0024	1·35E-16	0·0179	0·0064	5·47E-03	√	√
5	rs1933064	1	152301576	A	G	0·470	0·0157	0·0020	9·80E-15	0·0155	0·0054	4·04E-03	√	√
6	rs76798800	1	154994978	G	T	0·734	0·0122	0·0023	6·71E-08	0·0174	0·0062	4·83E-03	√	√
7	rs6672758	1	230303512	T	C	0·801	0·0176	0·0025	2·40E-12	0·0156	0·0067	1·90E-02	√	√
8	rs727857	2	58981967	G	A	0·389	0·0140	0·0021	1·05E-11	0·0109	0·0055	4·75E-02	√	√
9	rs1047891	2	211540507	C	A	0·684	0·0152	0·0021	1·18E-12	0·0127	0·0057	2·71E-02	√	√
10	rs2012736	2	234622379	C	A	0·919	0·0483	0·0037	1·16E-39	0·0384	0·0104	2·13E-04	√	√
11	rs6782190	3	85639672	G	A	0·352	0·0172	0·0021	1·45E-16	0·0207	0·0056	2·38E-04	√	√
12	rs705117	4	72608115	C	T	0·148	0·0334	0·0028	1·06E-32	0·0269	0·0074	2·94E-04	√	√
13	rs1352846	4	72617775	A	G	0·709	0·1935	0·0022	0	0·2222	0·0059	1·40E-310	√	√
14	rs78151190	6	25619007	A	C	0·871	0·0169	0·0030	1·39E-08	0·0187	0·0083	2·41E-02	√	√
15	rs75741381	7	100809458	C	G	0·852	0·0166	0·0028	4·15E-09	0·0214	0·0074	3·58E-03	√	√
16	rs12056768	8	116988527	T	G	0·417	0·0234	0·0020	6·44E-31	0·0177	0·0055	1·20E-03	√	√
17	rs77532868	10	88081438	T	C	0·054	0·0266	0·0044	1·57E-09	0·0281	0·0135	3·81E-02	√	√
18	rs12794714	11	14913575	G	A	0·578	0·0879	0·0020	0	0·0702	0·0054	1·22E-38	√	√
19	rs61891388	11	66079818	G	T	0·456	0·0126	0·0020	4·06E-10	0·0114	0·0054	3·40E-02	√	√
20	rs1660839	11	71094232	A	G	0·249	0·0293	0·0023	6·40E-37	0·0142	0·0062	2·30E-02	√	√
21	rs12803256	11	71132868	G	A	0·777	0·1042	0·0024	0	0·0839	0·0060	4·39E-44	√	√
22	rs12798050[b]	11	71223256	T	C	0·831	0·1100	0·0026	0	0·0348	0·0024	1·00E-47	√	√

© 2023 Sha T et al. *JAMA Network Open*.

23	rs72997623	11	75488054	A	C	0·085	0·0276	0·0036	1·25E-14	0·0200	0·0094	3·28E-02	√	√
24	rs1149605	11	76485216	C	T	0·170	0·0220	0·0027	1·31E-16	0·0210	0·0072	3·77E-03	√	√
25	rs10859995	12	96375682	T	C	0·417	0·0403	0·0020	1·05E-88	0·0366	0·0054	1·36E-11	√	√
26	rs8018720	14	39556185	G	C	0·177	0·0378	0·0026	1·26E-47	0·0409	0·0071	6·91E-09	√	√
27	rs261291	15	58680178	T	C	0·645	0·0274	0·0021	2·50E-39	0·0113	0·0056	4·41E-02	√	√
28	rs77924615	16	20392332	G	A	0·807	0·0166	0·0026	7·11E-11	0·0196	0·0067	3·55E-03	√	√
29	rs212100	19	48376995	T	C	0·164	0·0662	0·0027	1·61E-133	0·0194	0·0072	7·06E-03	√	√
30	rs10426	19	51517798	A	G	0·213	0·0257	0·0024	4·64E-26	0·0146	0·0065	2·51E-02	√	√
31	rs6123359	20	52714706	G	A	0·102	0·0342	0·0033	6·10E-25	0·0374	0·0094	7·08E-05	√	√
32	rs17216707	20	52732362	T	C	0·817	0·0376	0·0026	3·47E-46	0·0647	0·0066	2·02E-22	√	√
33	rs2585442	20	52737123	G	C	0·241	0·0357	0·0024	6·70E-51	0·0381	0·0064	1·96E-09	√	√
34	rs2762943	20	52790786	G	T	0·923	0·0457	0·0037	2·10E-34	0·0325	0·0144	2·41E-02	√	√
35	rs2074735	22	31535872	C	G	0·064	0·0278	0·0041	8·23E-12	0·0214	0·0105	4·27E-02	√	√
36	rs11591147	1	55505647	T	G	0·018	0·0451	0·0075	1·64E-09	-	-	-	×	√
37	rs2131925	1	63025942	G	T	0·356	0·0229	0·0021	3·61E-28	-	-	-	×	√
38	rs140371183	1	152098428	G	A	0·032	0·0870	0·0057	1·86E-53	-	-	-	×	√
39	rs12123821	1	152179152	T	C	0·048	0·0786	0·0047	2·55E-63	-	-	-	×	√
40	rs61816761	1	152285861	A	G	0·016	0·1232	0·0080	7·35E-53	-	-	-	×	√
41	rs10908419	1	154567699	G	A	0·510	0·0123	0·0020	5·81E-10	-	-	-	×	√
42	rs11264322	1	155087933	G	A	0·570	0·0094	0·0020	3·47E-06	-	-	-	×	√
43	rs10908465	1	155389688	T	C	0·267	0·0169	0·0022	6·12E-14	-	-	-	×	√
44	rs867772	1	220972343	A	G	0·315	0·0146	0·0022	1·14E-11	-	-	-	×	√
45	rs7604788	2	21190024	T	C	0·033	0·0337	0·0056	1·32E-09	-	-	-	×	√
46	rs541041	2	21294975	G	A	0·181	0·0155	0·0026	2·27E-09	-	-	-	×	√
47	rs1260326	2	27730940	C	T	0·607	0·0206	0·0020	4·41E-24	-	-	-	×	√

© 2023 Sha T et al. *JAMA Network Open*.

48	rs11127186	2	28881407	C	T	0·496	0·0109	0·0020	8·30E-08	-	-	-	×	√
49	rs2710651	2	63166379	G	A	0·472	0·0115	0·0020	9·51E-09	-	-	-	×	√
50	rs3849374	2	101443397	G	C	0·822	0·0161	0·0026	7·46E-10	-	-	-	×	√
51	rs7569755	2	118648261	A	G	0·291	0·0143	0·0022	1·18E-10	-	-	-	×	√
52	rs13060130	3	84440527	C	T	0·860	0·0150	0·0029	1·80E-07	-	-	-	×	√
53	rs9861009	3	141654685	C	T	0·728	0·0140	0·0023	4·86E-10	-	-	-	×	√
54	rs78649910	4	3482213	T	A	0·894	0·0212	0·0033	7·15E-11	-	-	-	×	√
55	rs4364259	4	15892159	A	G	0·202	0·0159	0·0025	2·16E-10	-	-	-	×	√
56	rs4616820	4	57745481	C	T	0·535	0·0123	0·0020	1·13E-09	-	-	-	×	√
57	rs35057908	4	69372082	T	A	0·431	0·0110	0·0020	5·31E-08	-	-	-	×	√
58	rs13104260	4	70348090	A	G	0·257	0·0073	0·0023	0·0014914	-	-	-	×	√
59	rs11732896	4	88287993	G	A	0·701	0·0160	0·0022	1·79E-13	-	-	-	×	√
60	rs28364331	4	100201295	G	A	0·018	0·0686	0·0075	4·19E-20	-	-	-	×	√
61	rs1229984	4	100239319	T	C	0·025	0·0451	0·0064	1·53E-12	-	-	-	×	√
62	rs10070734	5	87940026	C	T	0·710	0·0132	0·0022	1·82E-09	-	-	-	×	√
63	rs31612	5	108996643	T	C	0·826	0·0145	0·0026	4·15E-08	-	-	-	×	√
64	rs72834856	6	22801858	T	G	0·928	0·0250	0·0039	8·67E-11	-	-	-	×	√
65	rs28374650	6	32623367	C	T	0·756	0·0136	0·0023	5·44E-09	-	-	-	×	√
66	rs9476310	6	57767576	T	C	0·511	0·0118	0·0020	4·21E-09	-	-	-	×	√
67	rs9490317	6	121859499	C	T	0·446	0·0111	0·0020	3·95E-08	-	-	-	×	√
68	rs2248551	6	131924689	G	A	0·835	0·0234	0·0027	3·04E-18	-	-	-	×	√
69	rs10085881	7	21577960	T	C	0·718	0·0146	0·0022	7·83E-11	-	-	-	×	√
70	rs7784802	7	64015379	T	A	0·361	0·0138	0·0021	2·62E-11	-	-	-	×	√
71	rs6966728	7	104618318	C	T	0·537	0·0118	0·0020	8·01E-09	-	-	-	×	√
72	rs2346264	7	133536351	A	C	0·217	0·0139	0·0024	1·21E-08	-	-	-	×	√
73	rs34290760	8	9185179	C	G	0·971	0·0335	0·0059	1·70E-08	-	-	-	×	√

© 2023 Sha T et al. *JAMA Network Open*.

74	rs804281	8	11611865	G	A	0·584	0·0133	0·0020	4·72E-11	-	-	-	×	√
75	rs28692966	8	25892919	A	G	0·253	0·0148	0·0023	1·14E-10	-	-	-	×	√
76	rs2725371	8	30854033	G	A	0·698	0·0118	0·0022	5·84E-08	-	-	-	×	√
77	rs4738684	8	59393273	G	A	0·666	0·0124	0·0021	4·41E-09	-	-	-	×	√
78	rs13284054	9	107669073	C	T	0·118	0·0176	0·0031	2·07E-08	-	-	-	×	√
79	rs10887718	10	82042624	C	T	0·472	0·0111	0·0020	2·61E-08	-	-	-	×	√
80	rs3925446	10	91495322	A	G	0·199	0·0152	0·0025	1·09E-09	-	-	-	×	√
81	rs4418728	10	94839724	T	G	0·452	0·0110	0·0020	4·24E-08	-	-	-	×	√
82	rs61883501	11	13882754	A	C	0·967	0·0018	0·0055	0·749232	-	-	-	×	√
83	rs116970203	11	14876718	G	A	0·973	0·3769	0·0061	0	-	-	-	×	√
84	rs117576073	11	14912573	G	T	0·987	0·1472	0·0089	5·91E-62	-	-	-	×	√
85	rs78168201	11	70971149	T	C	0·014	0·0893	0·0086	5·18E-25	-	-	-	×	√
86	rs964184	11	116648917	C	G	0·868	0·0432	0·0029	1·09E-48	-	-	-	×	√
87	rs613808	11	116710968	G	A	0·720	0·0264	0·0022	3·40E-32	-	-	-	×	√
88	rs2847500	11	120114421	G	A	0·876	0·0219	0·0030	4·42E-13	-	-	-	×	√
89	rs12317268	12	21352541	A	G	0·849	0·0209	0·0028	6·19E-14	-	-	-	×	√
90	rs11182428	12	38526387	T	C	0·480	0·0125	0·0020	3·23E-10	-	-	-	×	√
91	rs1038165	12	68665940	T	C	0·583	0·0121	0·0020	2·31E-09	-	-	-	×	√
92	rs11108368	12	96386138	G	A	0·606	0·0038	0·0021	0·0669916	-	-	-	×	√
93	rs12372115	12	97982701	G	T	0·929	0·0218	0·0039	1·93E-08	-	-	-	×	√
94	rs73413596	12	111582630	C	T	0·074	0·0217	0·0038	1·41E-08	-	-	-	×	√
95	rs7149014	14	29802911	T	C	0·371	0·0129	0·0021	5·42E-10	-	-	-	×	√
96	rs12881545	14	101176212	C	G	0·673	0·0118	0·0021	2·91E-08	-	-	-	×	√
97	rs1800588	15	58723675	C	T	0·785	0·0329	0·0024	4·38E-42	-	-	-	×	√

© 2023 Sha T et al. *JAMA Network Open*.

98	rs55829990	15	63790642	T	C	0·656	0·0186	0·0021	9·12E-19	-	-	-	×	√
99	rs62007299	15	77711719	G	A	0·287	0·0133	0·0022	1·32E-09	-	-	-	×	√
100	rs325384	15	100229761	C	T	0·716	0·0142	0·0022	1·66E-10	-	-	-	×	√
101	rs17231506	16	56994528	C	T	0·677	0·0184	0·0021	5·45E-18	-	-	-	×	√
102	rs11076175	16	57006378	G	A	0·178	0·0230	0·0026	9·47E-19	-	-	-	×	√
103	rs4327060	16	72807438	C	T	0·946	0·0244	0·0044	2·92E-08	-	-	-	×	√
104	rs4575545	16	79755446	G	A	0·695	0·0156	0·0022	7·47E-13	-	-	-	×	√
105	rs11542462	16	82033810	G	A	0·866	0·0233	0·0029	1·27E-15	-	-	-	×	√
106	rs10454087	17	40735641	C	T	0·715	0·0135	0·0022	8·70E-10	-	-	-	×	√
107	rs2952289	17	66464414	T	C	0·798	0·0177	0·0025	1·18E-12	-	-	-	×	√
108	rs8091117	18	28919794	C	A	0·935	0·0264	0·0040	5·98E-11	-	-	-	×	√
109	rs4121823	18	47144223	T	A	0·155	0·0193	0·0028	3·83E-12	-	-	-	×	√
110	rs590215	18	57904088	C	T	0·734	0·0129	0·0023	1·04E-08	-	-	-	×	√
111	rs2037511	18	61366207	A	G	0·166	0·0181	0·0027	1·35E-11	-	-	-	×	√
112	rs142158911	19	11190534	A	G	0·115	0·0255	0·0031	4·79E-16	-	-	-	×	√
113	rs187429064	19	19380513	G	A	0·011	0·0648	0·0095	7·95E-12	-	-	-	×	√
114	rs3814995	19	36342212	C	T	0·688	0·0126	0·0021	5·18E-09	-	-	-	×	√
115	rs7412	19	45412079	T	C	0·082	0·0300	0·0036	1·36E-16	-	-	-	×	√
116	rs484195	19	45421877	A	G	0·384	0·0155	0·0021	1·37E-13	-	-	-	×	√
117	rs8113404	19	53065579	T	C	0·305	0·0122	0·0022	2·07E-08	-	-	-	×	√
118	rs11606	19	54658102	G	C	0·425	0·0120	0·0021	4·41E-09	-	-	-	×	√
119	rs2207132	20	39142516	G	A	0·967	0·0346	0·0056	5·56E-10	-	-	-	×	√
120	rs2229742	21	16339172	G	C	0·897	0·0251	0·0033	1·48E-14	-	-	-	×	√
121	rs6003456	22	23356100	T	A	0·765	0·0133	0·0024	1·95E-08	-	-	-	×	√

© 2023 Sha T et al. *JAMA Network Open*.

122	rs115621755	22	50853134	C	T	0·673	0·0124	0·0021	4·76E-09	-	-	-	×	√

[*] The summary statistics of serum 25(OH)D concentrations were extracted from 417,580 European-ancestry individuals in the UK Biobank study.

[†] The summary statistics of serum 25(OH)D concentrations were extracted from 79,366 European-ancestry individuals in the SUNLIGHT Consortium study.

BP: base-pair position, Chr: chromosome number; EAF: effect allele frequency; SE: standard error; SNV: single-nucleotide variant; vitamin D-GS: genetic instrument for serum 25(OH)D concentration; [a]SNV proxy in the SUNLIGHT consortium: rs1123571; [b]SNV proxy in the SUNLIGHT consortium: rs2186777.

© 2023 Sha T et al. *JAMA Network Open*.

eTable 2. Standard linear Mendelian randomization estimates for the associations of genetically predicted serum 25(OH)D with sarcopenia and its indices using 35 SNVs to instrument*

Outcome	Serum 25(OH)D (nmol/L) †	OR/beta (95%CI) ‡	*P*
Sarcopenia	<25·0	1.01 (1.00 to 1.02)	0·026
	25-49·9	1.00 (1.00 to 1.00)	0·799
	50·0-74·9	1.00 (0.99 to 1.00)	0·959
	≥75·0	1.00 (1.00 to 1.00)	0·573
Grip strength	<25·0	0.679 (0.627 to 0.730)	0·010
	25-49·9	0.109 (-0.062 to 0.280)	0.211
	50.0-74.9	0.135 (-0.031 to 0.302)	0.110
	≥75.0	0.089 (-0.196 to 0.374)	0.541
Appendicular lean mass index	<25.0	0.004 (-0.003 to 0.010)	0.262
	25-49.9	0.002 (0.001 to 0.004)	0.042
	50.0-74.9	0.000 (-0.002 to 0.002)	0.696
	≥75.0	-0.002 (-0.005 to 0.001)	0.290
Slow gait speed	<25.0	1.04 (1.01 to 1.06)	0.003
	25-49.9	1.01 (1.00 to 1.02)	0.004
	50.0-74.9	1.01 (1.00 to 1.01)	0.062
	≥75.0	1.00 (0.99 to 1.01)	0.990

*A total of 295,489 participants of unrelated European ancestry from the UK Biobank were included in the genetic analyses.

†Residual serum 25(OH)D.

‡per 10 nmol/L increase in serum 25(OH)D, OR for sarcopenia and slow gait speed, while a beta for grip strength and appendicular lean mass index. ORs/betas were estimated by two-stage least squares regression method with an adjustment for age, age-square, sex, birth location, assessment center, top 20 genetic principal components, genotyping array in both stages, and nuisance factors, which could affect serum 25(OH)D measurements, including the month in which blood sample was taken, fasting time before the blood sample was taken, and sample aliquots for measurement.

OR, odds ratio; CI, confidence interval.

© 2023 Sha T et al. *JAMA Network Open.*

eTable 3. Two-sample Mendelian randomization estimates for the association of genetically predicted serum 25(OH)D with sarcopenia and its indices using 35 SNVs to instrument*

Outcome*	MR Method	OR/beta (95%CI) †	*P*	$P_{Cochran's\text{-}Q}$ ‡
Sarcopenia	IVW	0.69 (0.38 to 1.25)	0.221	0.010
	Weighted median	0.75 (0.43 to 1.31)	0.307	
	MR Egger	0.59 (0.37 to 1.28)	0.189	0.008
	MR-PRESSO	0.69 (0.38 to 1.25)	0.230	
Grip strength	IVW	0.505 (0.011 to 1.000)	0.045	1.99e-66
	Weighted median	0.183 (0.017 to 0.350)	0.030	
	MR Egger	0.465 (-0.177 to 1.107)	0.165	7.01e-67
	MR-PRESSO	0.384(-0.203 to 0.889)	0.230	
Appendicular lean mass index	IVW	0.004 (-0.004 to 0.011)	0.328	1.35e-121
	Weighted median	0.001 (-0.002 to 0.003)	0.600	
	MR Egger	0.002 (-0.008 to 0.012)	0.651	2.81e-121
	MR-PRESSO	-0.002 (-0.008 to 0.004)	0.535	
Slow gait speed	IVW	0.92 (0.76 to 1.10)	0.350	2.39e-35
	Weighted median	1.05 (0.96 to 1.15)	0.240	
	MR Egger	1.06 (0.85 to 1.33)	0.608	2.89e-35
	MR-PRESSO	0.97 (0.78 to 1.21)	0.815	

*The summary statistics of sarcopenia, grip strength, and appendicular lean mass index were extracted from 324,976 unrelated European-ancestry individuals in the UK Biobank study, and the summary statistics of slow gait speed were extracted from 322,814 unrelated European-ancestry individuals in the UK Biobank study.

†per one unit increase in serum 25(OH)D, OR for sarcopenia and slow gait speed, while a beta for grip strength and appendicular lean mass index.

‡P for Cochran's Q statistic, which was used to assess the heterogeneity between SNVs. P for Cochran's Q statistic, which was used to assess the heterogeneity between SNVs. The detailed definition of Cochran's Q statistic was published elsewhere.[3]

MR, Mendelian randomization; SNV, single-nucleotide variant; OR, odds ratio; CI, confidence interval; MR-PRESSO, MR Pleiotropy RESidual Sum and Outlier; IVW, inverse-variance weighted.

© 2023 Sha T et al. *JAMA Network Open.*

eTable 4. Two-sample Mendelian randomization estimates for the association of genetically predicted serum 25(OH)D with sarcopenia and its indices using 35 SNVs to the instrument after excluding outliers

Outcome*	N SNVs	MR Method	OR/beta (95%CI) †	*P*	$P_{Cochran's\text{-}Q}$ ‡
Sarcopenia	30	IVW	0.78 (0.47 to 1.22)	0.284	0.737
		Weighted median	0.78 (0.45 to 1.37)	0.391	
		MR Egger	0.80 (0.43 to 1.49)	0.488	0.690
		MR-PRESSO	0.78 (0.50 to 1.23)	0.293	
Grip strength	15	IVW	0.101 (-0.246 to 0.448)	0.570	0.424
		Weighted median	0.060 (-0.380 to 0.501)	0.789	
		MR Egger	0.381 (-0.193 to 0.955)	0.216	0.453
		MR-PRESSO	0.101 (-0.246 to 0.448)	0.578	
Appendicular lean mass index	12	IVW	-0.002 (-0.006 to 0.003)	0.450	0.528
		Weighted median	-0.002 (-0.007 to 0.004)	0.581	
		MR Egger	-0.004 (-0.012 to 0.004)	0.389	0.467
		MR-PRESSO	-0.002 (-0.006 to 0.003)	0.446	
Slow gait speed	21	IVW	1.04 (0.88 to 1.22)	0.678	0.816
		Weighted median	1.03 (0.82 to 1.30)	0.779	
		MR Egger	1.11 (0.84 to 1.49)	0.469	0.789
		MR-PRESSO	1.04 (0.88 to 1.22)	0.628	

*The summary statistics of sarcopenia, grip strength, and appendicular lean mass index were extracted from 324,976 unrelated European-ancestry individuals in the UK Biobank study, and the summary statistics of slow gait speed were extracted from 322,814 unrelated European-ancestry individuals in the UK Biobank study.

†per one unit increase in serum 25(OH)D, OR for sarcopenia and slow gait speed, while a beta for grip strength and appendicular lean mass index.

‡P for Cochran’s Q statistic, which was used to assess the heterogeneity between SNVs. P for Cochran’s Q statistic, which was used to assess the heterogeneity between SNVs. The detailed definition of Cochran’s Q statistic was published elsewhere.[3]

MR, Mendelian randomization; SNV, single-nucleotide variant; OR, odds ratio; CI, confidence interval; MR-PRESSO, MR Pleiotropy RESidual Sum and Outlier; IVW, inverse-variance weighted.

© 2023 Sha T et al. *JAMA Network Open.*

eTable 5. Association of vitamin D-GS with potential confounders in the UK Biobank.

Instrument	Confounder	Beta†	SE	*P**
Vitamin D-GS	Body mass index	-0.00070	0.00038	0.067
	Smoking	0.00022	0.00035	0.526
	Alcohol intake	0.00005	0.00022	0.837
	Physical activity	0.00067	0.00041	0.122
	Townsend deprivation	-0.00106	0.00054	0.052

†Coefficients are in terms of an average-SNV increase in the allele score per unit/level increase in confounder. SE, standard error.

* P values have been adjusted for age, age-square, sex, genotyping array, birth location, assessment center and top 20 principal components of ancestry.

© 2023 Sha T et al. *JAMA Network Open.*

eTable 6. Functional blocks-related traits used in the sensitivity analyses.

SNV	Functional blocks related traits used in the sensitivity analyses*			
	Blood	Metabolic	Renal	Other
rs7522116	-	-	-	Intelligence, qualifications
rs7528419	Blood protein levels, progranulin levels	Angina pectoris, coronary artery disease, cholesterol, statin use	-	-
rs1933064	-	-	-	Atopic dermatitis
rs76798800	-	Various, incl. fat-free mass, birth weight, height		
rs6672758	Platelet count, red cell distribution	-	-	-
rs727857	-	Fat mass, BMI, impedance, weight	-	-
rs1047891	Various, incl. amino acid levels, white blood cell count, platelet count, hemoglobin, metabolite levels	Various, incl. weight, impedance, fat- free mass cholesterol	Creatinine, chronic kidney disease	
rs6782190	-	Fat free mass, BMI, impedance	-	Nervous feelings, alcohol intake, smoking, risk taking, number of children fathered
rs1352846	White cell count, granulocyte count, neutrophil count	-	-	-
rs78151190	Various, incl. hemoglobin, platelet count, reticulocyte count	Hematocrit, pulse, blood pressure	-	Disorders of mineral metabolism, Ferritin
rs75741381	-	Impedance	-	
rs12056768	-		-	Hair or balding pattern: pattern 4
rs12794714	-	Hip circumference	-	

© 2023 Sha T et al. *JAMA Network Open*.

rs61891388	-		-	Qualifications
rs12803256	-		-	Population differentiation
rs72997623	-	HDL cholesterol	-	
rs261291	-	Cholesterol, lipid metabolism	-	Age-related macular degeneration
rs77924615	-	Blood pressure	Glomerular filtration rate	-
rs212100	-	-	-	Cholelithiasis
rs17216707	-	-	Creatinine, glomerular filtration rate	-

*Functional blocks identified using trait associations identified through PhenoScanner V2.

© 2023 Sha T et al. *JAMA Network Open*.

eTable 7. Sensitivity analysis for non-linear Mendelian randomization analysis of serum 25(OH)D on sarcopenia risk.

Excluded functional block	SNVs associated with the functional block	$P_{non-linear}$*			
		Sarcopenia	Grip strength	Appendicular lean mass index	Slow gait speed
Blood traits	rs1047891, rs1352846, rs6672758, rs72997623, rs7528419, and rs78151190	0.038	0.001	0.004	0.004
Metabolic	rs76798800, rs78151190, rs77924615, rs261291, rs727857, rs1047891, rs12794714, rs72997623, rs7528419, rs75741381, and rs6782190	0.097	0.016	0.004	<0.001
Renal	rs1047891, rs17216707 and rs77924615	0.025	0.008	0.002	<0.001
Other	rs6782190, rs61891388, rs12803256, rs1047891, rs212100, rs7522116, rs78151190, rs261291, rs12056768, and rs1933064	0.161	<0.001	<0.001	<0.001

*Likelihood ratio test comparing the besting-fitting fractional polynomial model of degree 1 against the linear model

© 2023 Sha T et al. *JAMA Network Open*.

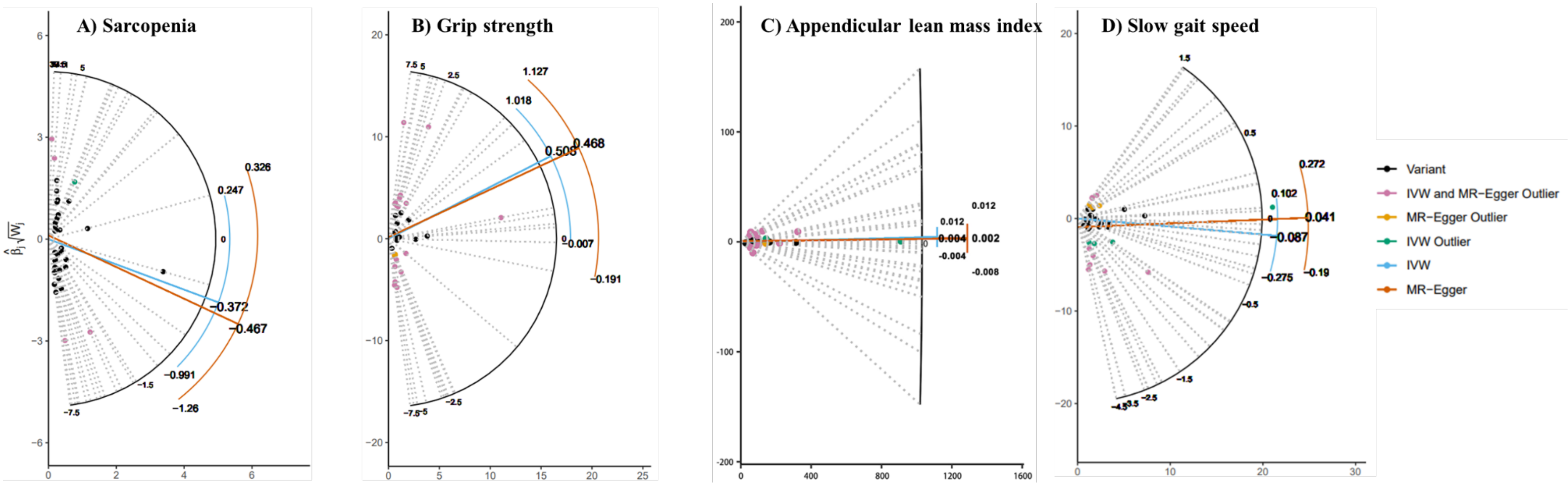

eFigure 1. Radial Mendelian randomization plots for serum 25(OH)D and sarcopenia and its indices using 35 SNVs to the instrument.

© 2023 Sha T et al. *JAMA Network Open*.

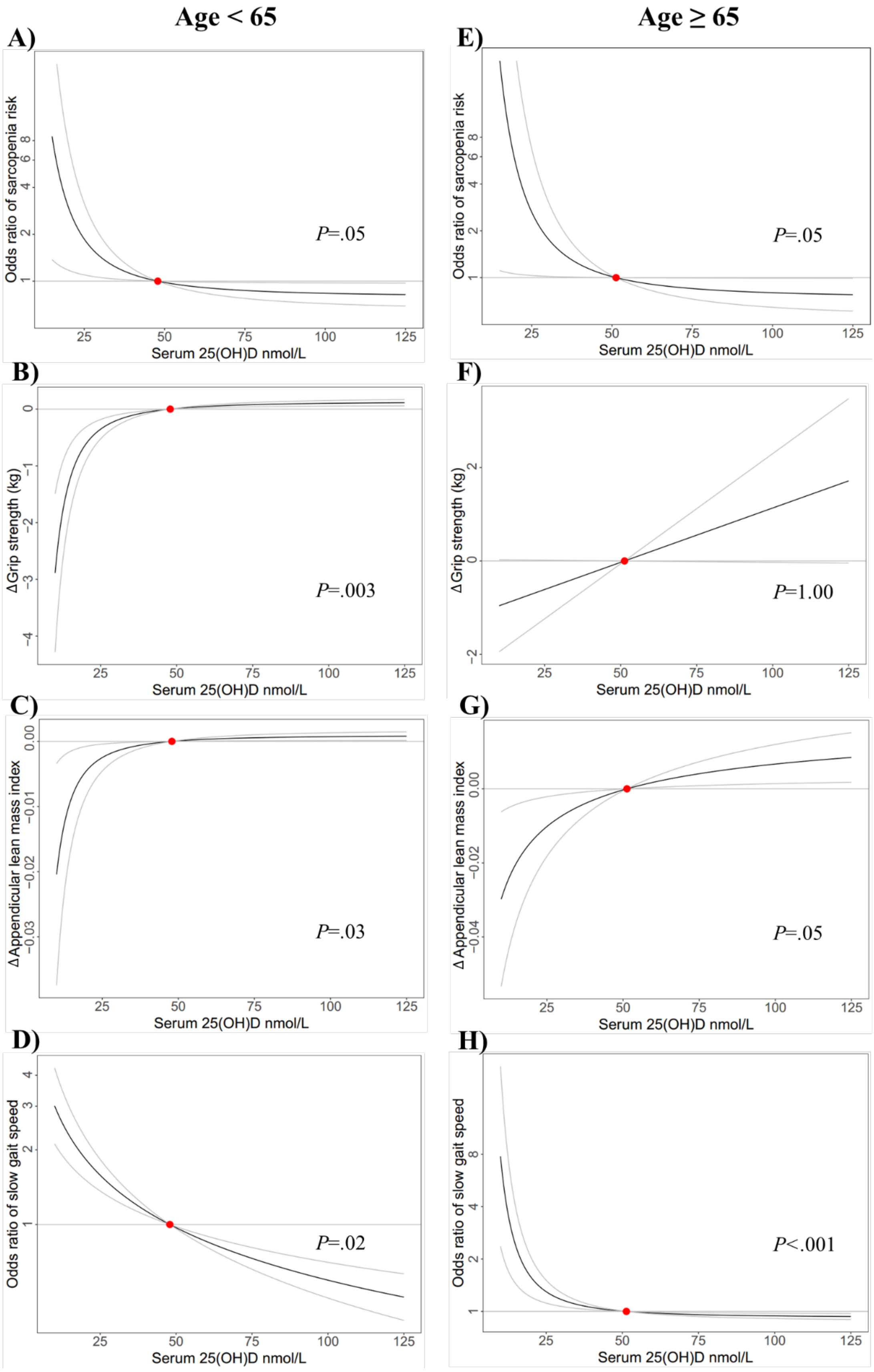

eFigure 2. Age-stratified non-linear Mendelian randomization analyses of genetic associations of serum 25(OH)D with sarcopenia and its indices,

© 2023 Sha T et al. *JAMA Network Open.*

(A) and (E) sarcopenia, (B) and (F) grip strength, (C) and (G) appendicular lean mass index, and (D) and (H) slow gait speed. The left panels (age <65 years old) and the right panels (age ≥65 years old). The red dot represents the reference point of serum 25(OH)D of 50nmol/L. The gray lines represent the 95% confidence intervals. The adjustment included age, age-square, sex, assessment center, birth location, top 20 genetic principal components, genotyping array in both stages, and nuisance factors, which could affect serum 25(OH)D measurements, including the month when the blood sample was taken, fasting time before the blood sample was taken, and sample aliquots for measurement.

© 2023 Sha T et al. *JAMA Network Open*.

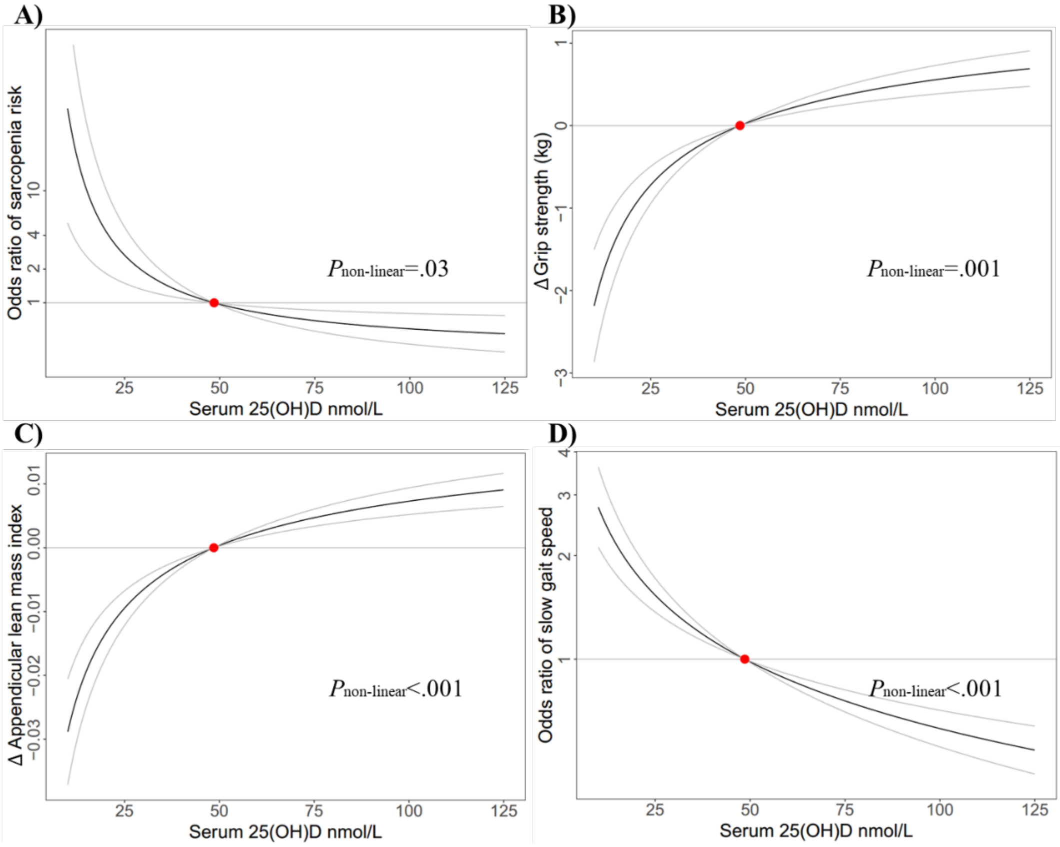

eFigure 3. Genetic associations of serum 25(OH)D with sarcopenia and its indices using 122 SNVs to instrument, (A) sarcopenia, (B) grip strength, (C) appendicular lean mass index, and (D) slow gait speed. The red dot represents the reference point of serum 25(OH)D of 50nmol/L. The gray lines represent the 95% confidence intervals. The adjustment includes age, age-square, sex, assessment center, birth location, top 20 genetic principal components, genotyping array in both stages, and nuisance factors, which could affect serum 25(OH)D measurements, including month in which blood sample was taken, fasting time before blood sample was taken, and sample aliquots for measurement.

© 2023 Sha T et al. *JAMA Network Open.*

eReferences

1. Burgess S, Thompson SG. Interpreting findings from Mendelian randomization using the MR-Egger method. *Eur J Epidemiol*. 2017;32(5):377-389. doi:10.1007/s10654-017-0255-x
2. Verbanck M, Chen CY, Neale B, Do R. Detection of widespread horizontal pleiotropy in causal relationships inferred from Mendelian randomization between complex traits and diseases. *Nat Genet*. 2018;50(5):693-698. doi:10.1038/s41588-018-0099-7
3. Greco MF, Minelli C, Sheehan NA, Thompson JR. Detecting pleiotropy in Mendelian randomisation studies with summary data and a continuous outcome. *Stat Med*. 2015;34(21):2926-40. doi:10.1002/sim.6522
4. Staley JR, Burgess S. Semiparametric methods for estimation of a nonlinear exposure-outcome relationship using instrumental variables with application to Mendelian randomization. *Genet Epidemiol*. 2017;41(4):341-352. doi:10.1002/gepi.22041
5. Lawlor DA, Harbord RM, Sterne JA, Timpson N, Davey Smith G. Mendelian randomization: using genes as instruments for making causal inferences in epidemiology. *Stat Med*. 2008;27(8):1133-63. doi:10.1002/sim.3034

© 2023 Sha T et al. *JAMA Network Open.*
