# Supplementary material for: Genetic Variants, Serum 25-Hydroxyvitamin D Levels, and Sarcopenia: A Mendelian Randomization Analysis
Source: JAMA Netw Open. 2023 Aug 30;6(8):e2331558. doi: 10.1001/jamanetworkopen.2023.31558 (PMC10469287; doi:10.1001/jamanetworkopen.2023.31558)
Supplement: Supplement 2. — Data Sharing Statement [file jamanetwopen-e2331558-s002.pdf]

Data Sharing Statement

Sha. Genetic Variants, Serum 25-Hydroxyvitamin D Levels, and Sarcopenia. *JAMA Netw Open*. Published August 30, 2023. doi:10.1001/jamanetworkopen.2023.31558

Data
Data available: No

Additional Information
Explanation for why data not available: Data used in the study is publicly available at reported resources. Primary data from the UK Biobank resource are accessible upon application (https://www.ukbiobank.ac.uk/). The complete Genome-wide association study summary-level data for serum 25(OH)D concentration are available at https://www.ncbi.nlm.nih.gov/pmc/articles/PMC7118120/, https://www.ncbi.nlm.nih.gov/pmc/articles/PMC5772647/.
